# Photodynamic Therapy with Verteporfin for Chronic Central Serous Chorioretinopathy: A Review of Data and Efficacy

**DOI:** 10.3390/ph13110349

**Published:** 2020-10-29

**Authors:** Pierluigi Iacono, Stefano Da Pozzo, Monica Varano, Mariacristina Parravano

**Affiliations:** 1 IRCCS-Fondazione Bietti, Via Livenza 3, 00198 Rome, Italy; m.varano@mclink.it (M.V.); mcparravano@gmail.com (M.P.); 2 TSRetina, 34121 Trieste, Italy; stefanodapozzo@yahoo.it

**Keywords:** central serous chorioretinopathy, photodynamic therapy with verteporfin, subthreshold laser treatment, antagonists of mineralocorticoid receptors, anti-VEGF drugs, transpupillary thermotherapy

## Abstract

Central serous chorioretinopathy represents the fourth most frequent retinal disorder, occurring especially in young age. Central serous chorioretinopathy is mainly characterized by macular serous retinal detachment and although the clinical course moves frequently toward a spontaneous resolution, the subretinal fluid may persist for a long time, thus evolving to the chronic form, and leading to a potential damage of the retinal pigment epithelium and to photoreceptors. The photodynamic therapy with verteporfin plays an important role in the armamentarium among the many therapeutic options employed in this complex retinal disorder. In this review, the authors aim to summarize data of efficacy and safety of PDT focusing especially on mechanisms of action of the PDT and providing comparative outcomes with the alternative therapeutic approaches, including especially the subthreshold laser treatment.

## 1. Introduction

Central serous chorioretinopathy (CSC) is a retinal disorder with a high prevalence in the population, ranking in fourth place, involving mainly male subjects, aged between 30 and 50 years. The dominant morphological feature is the central sensory neuroretinal detachment (NSD) which mainly involves the macular area and the posterior pole. The symptoms associated at the onset are characterized by a visual acuity reduction, central visual distortion and the presence of a relative scotoma with possible association of dyschromatopsia, micropsia and reduced contrast sensitivity [1,2,3,4].

For many years, angiography with fluorescein and indocyanine green has played a primary role in the diagnosis and recognition of the role of the retinal pigment epithelium (RPE) and choroidal circulation in the pathogenetic mechanism of CSC. However, it is with the advent of the structural optical coherence tomography (OCT) and OCT Angiography (OCTA) examinations that the precise determination of the anatomical alterations of the retina, the quantification of the extent of NSD and especially the assessment of the vascular and flow alterations of the choroidal laminae have become possible.

The complex pathogenetic mechanism that underlies CSC makes it difficult to achieve a precise classification of a retinal disorder which appears in multiple patterns of manifestation, shows a course not always predictable, with a spontaneous resolution of NSD varying between few weeks and 1–2 years. Most cases of CSC resolve spontaneously without treatment; however, patients with chronic NSD or with recurrences may evolve toward RPE atrophy and neurosensory retinal damage, leading to a permanent visual loss. The natural history of spontaneous resolution in majority of patients makes it difficult to evaluate the effectiveness of the therapeutic treatments available, especially those aimed at treating the chronic form CSC (cCSC).

Current off-label therapeutic options for cCSC mainly include the photodynamic therapy with verteporfin (PDT) and subthreshold laser treatment (STLT); more recently, therapies with mineralocorticoid antagonist drugs and anti-vascular endothelial growth factor (anti-VEGF) drugs have been applied, and finally some studies exploring the use of oral drugs such as mifepristone, ketoconazole, rifampin, finasteride, methotrexate, oral acetazolamide are also available [5].

The aim of this review is to present the effects and mechanisms of action of PDT in the cCSC, to evaluate the efficacy of the treatment and the protocols currently used and to examine the comparative studies with the other therapeutic options available.

## 2. Results

### 2.1. Mechanism of Action and Early Changes after PDT Administration

In 2003, the first case reports and small case series on the off-label use of verteporfin PDT in patients with cCSC were published independently and almost simultaneously [6,7,8]. The hypotheses supported by the authors seemed to converge on the opportunity to act on the altered choroidal hyperpermeability. In particular the authors argued that verteporfin PDT promotes a choriocapillaris hypoperfusion, possibly by mean of a direct action on the choriocapillaris endothelium and the consequent choriocapillaris occlusion, with a secondary improvement of the choroidal congestion, a reduction of vascular hyperpermeability and of the extravascular leakage.

Verteporfin is a photosensitizer and binds to vascular endothelial cells by means of the lipoprotein receptors located on the plasma membrane. The activated form of the verteporfin generates reactive oxygen species capable of inducing oxidative damage to the endothelial wall with release of procoagulant factors, platelet aggregation, clot formation and vasoconstriction [9]; as a consequence, the PDT would induces a choriocapillaris damage and vascular remodelling thus decreasing choroidal hyperpermeability and, also, would restore the blood retinal barrier at the level of the RPE finally resulting in resolution of NSD [10].

The preliminary results presented seemed to be immediately comforting with an improvement in visual function of at least 1–2 lines in 30–80% of cases, a resolution of the NSD in 60% of the subjects and a marked reduction of the choroidal hyperpermeability in almost all cases [6,7,8].

The effects of standard PDT appear rapidly after administration of the treatment with a variable effect on visual function and in the anatomical response, dispelling any doubts on the direct and immediate effect of PDT on the reabsorption of subretinal fluid, a condition that still persists in other therapies employed for cCSC including mineralocorticoid antagonist and anti-VEGF drugs.

Over the first few days after the treatment, three different patterns of early anatomic response to PDT treatment may be identified: the NSD can reduce consistently or resolve completely (63%), may increase (16%) or show a wide fluctuation (21%) [11]. Visual acuity is variably affected. It may show stabilization or reduction when associated with partial or complete resolution of the NSD; it is substantially stable when associated with an increase in subretinal fluid collection, however it is normally accompanied by an increase in metamorphoses; finally, in the pattern characterized by wide and fast fluctuation in the height of the NSD, the visual acuity may decrease or improve. The transient reduction in visual acuity could be related to the abrupt reattachment of the neurosensory retina, with incomplete rearrangement of the photoreceptor and RPE layers or probably could be correlated with a deterioration of retinal neuronal function, as reflected by electroretinography assessment [12,13]. In the majority of cases, there is a reduction in retinal sensitivity (RS) on the microperimetric examination that cannot be correlated with the different morphological patterns of variation of the NSD. Finally, the standard PDT induce a significant reduction in the central choroidal thickness detectable in approximately 95% eyes.

Van Dijk et al. reported data on 1-week changes in subjects with cCSC and receiving PDT with half-dose of verteporfin [14]. In this group a greater proportion of patients showed a visual acuity decline, a lower proportion of subjects achieved a NSD resolution, RS showed a substantial stabilization as well as the central choroidal thickness. The comparison between the two studies would suggest that PDT affects visual acuity regardless of dosage and independently of the spot PDT diameter, as well as the site of application. On the contrary, it appears evident that PDT with the standard protocol is responsible for a fast resolution of NSD, for the greater and rapid reduction in the choroidal thickness and for the reduction in RS. The early changes of the central choroidal thickness and of the NSD after half-dose and half-fluence PDT seems to be also corroborated in the studies of Maruko and Alkin, respectively [15,16].

### 2.2. Standard Photodynamic Therapy and Adapted Protocols

To evaluate the effectiveness of PDT in cCSC, we operated a literature screening to find clinical studies with at least 12 months of follow-up. Special focus was direct to studies reporting data on visual acuity, RS, percentage of resolution of NSD, number of recurrences, beneficial effects of retreatment, complications and side effects. Among 277 selected manuscripts dealing with cCSC and PDT, 55 were taken into account for the selected parameters; 9 had a prospective design.

#### 2.2.1. Standard PDT

Standard PDT was employed with setting as per TAP protocol in AMD complicated by choroidal neovascularization (CNV) (Treatment of age-related macular degeneration (AMD) with photodynamic therapy (TAP) Study Group). Shortly, PDT was performed by using the normal dose of 6 mg/m^2^ of verteporfin (Visudyne^®^; Novartis, Basel, Switzerland). The photosensitizer was infused through intravenous access over 10 min. Fifteen minutes after the start of the infusion, the laser light was applied for 83 s to the area of choroidal hyperpermeability to result in a light exposure of 50 J/cm^2^ from a 689-nm laser system (Carl Zeiss, Dublin, CA, USA).

Overall, follow-up ranged between 1 and 5 years with 8/13 studies with 1-year follow-up and 4/13 with prospective design [10,11,17,18,19,20,21,22,23,24,25,26,27] (Table 1).

The BCVA showed a significant mean final improvement in all studies with a mean visual acuity gain in a range of 8–14 letters, 0.15–0.40 Logmar, 0.07–0.23 decimal unit. Despite it is assumed BCVA is not the best parameter to represent the change and the effects of treatment in the visual function when dealing with cCSC, the RS was investigated in only two studies. Reibaldi et al. reported a statistically significant improvement of 2.5 dB and a final mean value of 14.4 dB; similarly, in the Iacono’s study, the standard PDT lead to an improvement of 2.69 dB and a final value of 14.1, over 1-year follow-up [11,22]. It is worth of note that the two studies demonstrated that RS achieved a mean final value very similar to the values obtained in untreated subjects with spontaneous resolution of cCSC [28].

It is very interesting the broad consensus found in the analysis of the percentage of NSD resolution, number of treatments and number of NSD recurrences. A complete resolution of the NSD was achieved in 90–100% of cases at 1-year examination in 11/13 studies (85%) with 8/13 studies (61%) reporting 100% NSD resolution at the last visit; in 5 of these 11 studies, a single PDT session was administered at the baseline and no recurrence was registered during the follow-up. Also in studies with longer follow-up [23,25], the NSD achieved a complete resolution in 100% of patients with 1 or 2 recurrence occurring between the second and seventh year of the study period. Finally, the mean value of the PDT does not exceed 1.3 treatment and no more of two additional PDT sessions were administered in two patients and no more of a total of 4 patients showing recurrence did not achieve NSD resolution despite re-treatment.

Complications of PDT in cCSC were rarely reported; in detail no serious adverse event related to PDT administration was described. CNV occurred in a total of 4 cases [10,20], secondary RPE atrophy was described in the 7% of cases by Noh [18], and RPE hyperplasia was identified in 9/72 subjects by Moreno [20]. In the other studies, no complication related to PDT session or PDT effects was reported in 4 studies, whereas no specific data was presented in 6 studies.

The preliminary conclusions suggest the standard PDT protocol is effective in inducing resolution of NSD and leading to a significant improvement in visual acuity. However, the application of standard PDT in cCSC is not without drawbacks, including the onset of RPE hyperplasia or atrophy, transient choriocapillary ischaemia and CNV.

#### 2.2.2. Half-Dose PDT (HD-PDT), Half-Fluence PDT (HF-PDT), Half-Time PDT (HT-PDT)

With the aim of reducing side effects related to standard PDT, the treatment setting parameters were modified in many studies by reducing verteporfin dose, fluence, laser treatment times and more rarely shortening the time between verteporfin injection and laser application.

Among modified PDT regimen, half-dose—3 mg/m^2^ of verteporfin—is definitely the most frequent adopted protocol for cCSC. Studies are predominantly retrospective in the design with prospective studies no longer than 2 years follow-up [29,30,31,32,33,34,35,36,37,38,39,40,41] (Table 2).

The efficacy of HD-PDT in term of BCVA is generally very positive with a visual acuity improvement between 0.12 and 0.39 Logmar unit. Only Fujita evaluated prospectively the long-term effects of HD-PDT on RS; a mean improvement of 5.23 dB could be registered at 1-year follow-up.

The HD-PDT achieved a complete NSD resolution in 75–100% of cases with only 4/13 studies (30%) reporting 100% NSD resolution. Compared with standard PDT, recurrences of NSD in HD-PDT studies were more frequent with 10/13 studies (77%) reporting a proportion of NSD recurrences in a range of 1–12%. As a consequence, in 9/13 studies, a second HD-PDT session was performed. Complications related to HD-PDT including RPE atrophy and CNV were rarely reported in the study with 1-year follow-up. Of interest, the two studies with the longest follow-up of Lai et al. [39] (36 months) and of Tseng et al. [40] (55 months) reported a similar value complication of about 5% (RPE atrophy, RPE rip, subretinal fibrosis, CNV). No data regarding complication related to HD-PDT was presented in 38% of studies.

Only few studies with at least one-year follow-up reported on the effects of modified fluence PDT for cCSC [41,42,43,44,45] (Table 3).

Also in the studies with HF-PDT, the BCVA improved significantly in a range of 0.11–0.37 Logmar, with one study reporting a 5 letter gain and Inoue reporting 1 decimal unit improvement. Unfortunately, no study examined the effect on RS. Most of the studies set the fluence to a value of 50% of standard PDT; Park et al. compared three different fluences, 30-40-50% [42]. In 4/5 studies (80%), the NSD resolved completely in 100% of cases with 50% fluence. Fluence at 30% and 40% obtained, respectively, a complete NSD resolution in the 80% and 94% of subjects.

NSD recurrences were described in all studies with HF-PDT (fluence 50%) where the value of 6–24% of cases was registered. A higher proportion of recurrences equal to 50% and 25% was identified in the subgroups with fluence 30% and 40%, respectively. In parallel, a second treatment was administered in all cases of recurrences. Of note, the study of Park et al. [42], clearly demonstrated that while the value of fluence is reduced there is an increase in the number of NSD recurrences and a reduction in the success rate of resorption of subretinal fluid in NSD. Of further interest, RPE atrophy was never reported in all studies with just 1 case of CNV occurrence in the Inoue’s study.

Only few retrospective studies with mean 12-months follow-up applied the half-time protocol, halving the exposure time to reduce the total energy to one-half of that of standard PDT [46,47]. Iwase et al. described the effect of HT-PDT in 22 subjects with cCSC over 24-months follow-up. The BCVA increased at the last visit by 0.14 Logmar and a significant improvement of 5.50 dB on RS was obtained (MAIA perimeter). Just one HT-PDT session was administered to obtain a complete reabsorption of the subretinal fluid in the 100% of cases with only 1 case of NSD recurrence; simple observation was adopted for this single case who evolved subsequently to spontaneous resolution. No complication related to PDT administration or over the study course was reported.

Finally, the different adapted treatment strategies were evaluated in comparative studies [48,49,50,51,52,53] (Table 4).

Mean follow-up of studies ranged between 1 and 3 years with just one study with prospective design. Most of them compared standard PDT to HF-PDT, and some HD-PDT vs. HT-PDT or HD-PDT vs. HF-PDT. Overall, final outcomes did not seem to be different from results of studies considering a single treatment arm. BCVA improved significantly in all studies and in all subgroups with a mean improvement between 0.111 and 0.30 Logmar. RS was never evaluated. Standard PDT achieved a complete NSD resolution in all but one study. In this specific study, Reibaldi et al. registered 2 recurrences [49]. HF-PDT achieved similar proportion of NSD resolution; however, recurrences occurred more frequently although the absolute number of recurrences was very low. Complications over the follow-up occurred rarely with 3 case of RPE atrophy and 1 case of CNV in standard PDT subgroups and just 2 cases of RPE atrophy in the study of Son, over a follow-up of 3 years [48].

Comparative trials evaluating half-dose versus half-time or half-fluence showed final similar outcomes with regard to the proportion of complete NSD resolution, varying from 91% to 100%; however more frequent recurrences were registered in these studies, thus requiring a second PDT session. Complications were rare with 2 cases of CNV reported specifically in the HD-PDT subgroup in the study of Peng [46].

### 2.3. PDT vs. Subthreshold Laser Treatment

There is only a limited number of comparative studies that have evaluated the results of PDT in comparison to subthreshold laser treatment (STLT) in prospective trials or retrospective studies [54,55,56,57,58,59,60] (Table 5).

The STLT, otherwise called non-damaging laser or sublethal laser, would selectively act on the activity of the retinal pigment epithelium by promoting the migration and proliferation of new cells and activating the release mechanism of the heat shock proteins (HSP). Upon resumption of RPE activity and following the activation of HSP, a progressive but slow reabsorption of the subretinal fluid would occur.

Most of the retrospective studies analyzed the effects of STLT with 577nm wavelength versus HF-PDT or HD-PDT. The results appear to be positive with complete resolution of NSD, ranging from 36% to 92% over a 2–12 month follow-up, and broadly similar to resolution rates in the PDT groups. However, there are substantial differences in the protocol application of the STLT with regards to the treatment area. Alternatively, the studies’s settings included the treatment of the areas of leakage recognized as hotspots in fluorescein angiography (FA), the areas of hyperfluorescence on ICGA and the corresponding areas of leakage on FA were treated, or the edematous areas recognized in the OCT examination. On the contrary, the authors seem to apply a common laser setting with duty cycle 5%, spot diameter included between 100–200 microns, 200 ms in duration and with confluent spots and selecting the power after titration in a healthy area outside the area to be treated.

The results also appear positive for the recovery of visual function in terms of visual acuity and reduction of central retinal thickness. The authors seem to conclude positively for the effectiveness of the STLT, also underlining the easy administration and laser treatment management as well as the relative advantages and lower costs compared to PDT therapy.

Two prospective randomized clinical trials with short-term follow-up have recently been published. Ho et al. analyzed data from 33 cCSC patients in a 6-month follow-up single-center study [59]. Eighteen subjects were assigned to STLT and 15 to HD-PDT. A significant improvement in visual acuity and central retinal thickness was demonstrated in both groups with no significant difference between the two groups in the baseline and final values. Unfortunately, percentage of complete resolution of the NSD at 6-month examination was not reported. However, the primary outcome of the study was the evaluation of signal changes in the choriocapillary flow deficit and in the volumes of the choroidal thickness, highlighting the differences in regard to the two treatments.

The quantitative parameter for the flow signal deficits assessed by OCT angiography, namely the mean total signal area of the flow deficit, was similar in both groups at baseline (STLT 0.319 ± 0.301 vs. PDT 0.231 ± 0.203 mm^2^), improved more rapidly in the PDT group and reached a similar final mean value in the two groups at six months (STLT 0.111 ± 0.0.061 vs. PDT 0.0.98 ± 0.057 mm^2^) with a final percentage of improvement on the mean values, compared to the baseline, of 65% in STLT group and 57% in the PDT group. The analysis of the central macular volume showed a progressive and statistically significant reduction in the mean values in the PDT group while the STLT group showed a progressive increase although not statistically significant; the authors did not provide an interpretation of this phenomenon which appears new and partially dissenting from the data available in the literature [61,62].

The PLACE trial (half-dose photodynamic therapy versus high-density subthreshold micropulse laser treatment in patients with chronic central serous chorioretinopathy) represents the largest multicentre, randomized, controlled, open-label study that has evaluated the anatomical and functional efficacy of HD-PDT and STLT treatment in a short-term follow-up [60]. The trial study was designed as a superiority study of HD-PDT over STLT with a 22% greater expectation of success in the HD-PDT arm. 179 patients were enrolled, 89 patients in the PDT group and 90 patients in the STLT group. At the final visit at 7/8 months examination, both groups showed a significant improvement in visual acuity and in the NEI-VFQ25 score without significant differences between the two groups. In particular, the HD-PDT group showed a higher percentage of complete resolution of the subretinal fluid (67.2% vs. 28.8%) and a more significant improvement in central RS, compared with the STLT group (+3.24 dB vs. 1.38 dB).

Finally, the authors concluded that Half-dose PDT is superior to STLT for treating cCSC, leading to a significantly higher proportion of patients with complete resolution of SRF and functional improvement.

### 2.4. PDT vs. Transpupillary Thermotherapy

Transpupillary thermotherapy (TTT) is a technique in which low heat levels are delivered through the pupil using a modified diode laser. The moderate increase in local temperature would be able to free the heat shock proteins with a subsequent activation of the mechanisms of migration, transformation and re-proliferation of the RPE together with phenomena of vascular thrombosis of the choriocapillary with a partial reduction of the blood flow, by acting in essence with a mixed mechanism similar to PDT and to the subthreshold laser treatment. To the best of our knowledge, only 2 comparative studies have been published. Manayath et al. presented the outcomes of a comparison between half-fluence PDT versus graded subthreshold TTT (by using an 810 nm long pulse diode laser) in a prospective study recruiting 42 subjects and during a course study of 6-months follow-up [63]. At the final visit, BCVA show a significant improvement by 0.26 Logmar and 0.24 Logmar, in the HF-PDT and TTT groups, respectively. HF-PDT and TTT achieved a complete resolution of NSD in 85% and 77%, respectively, whereas 15% in the HF-PDT group and 23% in the TTT group showed a persistent NSD despite additional re-treatments. At the 6-month follow-up none of the patients developed any ocular side effects related to the treatment including RPE atrophy, macular scar, or CNV. In the other prospective study, Russo et al. compared HD-PDT versus TTT (by using a 689 nm laser treatment and delivering 95 J/cm^2^ via an intensity application of 805 mW/cm^2^ over 118 s) in 40 patients [64]. In spite of a better value of visual acuity recorded in the HD-PDT group at baseline, both groups demonstrated a significant improvement in BCVA with a gain of 0.25 Logmar in the HD-PDT group and 0.34 in the TTT group, and a final value not different between the two groups (*p*: 0.28). At 10-month evaluation, NSD completely resolved in all patients with no recurrences registered during the follow-up. With regard to the safety profile, none of the patients developed any ocular or systemic adverse events associated with the treatments. In both studies, a faster resolution of the NSD could be observed in the HD/HF-PDT groups confirming the mechanism of action of PDT; by contrast, eyes included in the TTT group showed a progressive reabsorption of the subretinal fluid taking more time and acting more similarly with the mechanism of action of subthreshold laser treatment.

### 2.5. PDT vs. Mineralcorticoids

CSC has still some unclear aspects in its pathogenesis, including the potential role of corticosteroids, especially glucocorticoids and mineralocorticoids. In particular, a disorder in glucocorticoid metabolism and an abnormal aldosterone/mineralocorticoid (MR) receptor pathway has been hypothesized; such receptor is expressed in the neurosensorial retina, RPE and choroid and a defect of activity could lead to choroidal vasodilation and subretinal fluid accumulation. The preliminary study by Zhao et al. demonstrated rapid resorption of subretinal fluid in 2 subjects with unresolved CSC after oral treatment with eplerenone, MR antagonists used in the treatment of primary aldosteronism and hypertension [65]. Lee et al. reported a first comparative study with 3-month follow-up with oral spironolactone treatment versus HD-PDT in patients with nonresolving CSC [66]. BCVA improved significantly only in the HD-PDT group whereas complete NSD regression was observed at three months in 38.9% patients receiving spironolactone and in 56.5% of patients in the HD-PDT group. More recently, the study of Kim et al. presented data on a retrospective comparative study on oral spironolactone and HD-PDT with one-year follow-up [67]. Fifty patients were evenly distributed between the two groups. Visual acuity improved significantly with both treatments with no difference between the two groups at the baseline and at 12-month visit. A complete NSD resolution during the follow-up period was observed in 18/26 subjects (69.2%) in the spironolactone group and in 21/24 subjects (87.5%) in the HD-PDT group. Despite a similar proportion of NSD resolution, the spironolactone group showed a higher rate of recurrences after drug discontinuation -66.7%- compared with the HD-PDT group, 14.3%. No ocular or systemic serious adverse event was registered in both groups during the 1-year study.

### 2.6. PDT vs. Anti-Vascular Endothelial Growth Factor

Although there is no evidence of pathological and increased intravitreal levels of vascular endothelial growth factor or other intraocular cytokines, there is a belief demonstrated by some authors that anti-VEGF drugs can reduce choroidal vascular hyper-permeability by modulating VEGF levels.

Bae et al. carried out a prospective randomized clinical trial comparing low-fluence PDT with ranibizumab in 32 patients with cCSC in a 12-month follow-up study [68]. The patients underwent a single session of HF-PDT or three consecutive monthly intravitreal ranibizumab injections. Study design included also a rescue treatment from three months on; if the NSD persisted or recurred after primary treatment, the patient could switch from HF-PDT to ranibizumab and vice versa. At months 12, BCVA improved significantly from baseline by 0.26 and 0.19 Logmar in the low-fluence PDT group and ranibizumab group, respectively, with no significant difference between the two groups. Despite a similar change in BCVA over the follow-up, the authors reported a remarkable superiority of HF-PDT over ranibizumab on proportion of eyes with complete NSD resolution with 16 eyes (88.9%) in the HF-PDT group and two eyes (12.5%) in the ranibizumab group. In parallel, a significant greater proportion of patients in the ranibizumab group (11 eyes [68.8%]) met the criteria for rescue treatment compared with the HF-PDT group (two eyes [11.1%]; *p*: 0.001). Indocyanine green angiography examination confirmed a marked reduction in choroidal hyperpermeability in the HF-PDT group compared with ranibizumab group (88.9% vs. 0) providing clear evidence of an overall superiority of HF-PDT over ranibizumab. No serious adverse events related to the HF-PDT or ranibizumab were registered. Semeraro et al. reported data on comparison between HF-PDT and intravitreal bevacizumab in a prospective study with a mean follow-up of 9 months [69]. Although the macular thickness decreased significantly during the follow-up period in both groups, no data on proportion of NSD resolution was provided. Bevacizumab efficacy was also evaluated in comparison to standard PDT in short term follow-up study by Lee et al. Also in this study, BCVA course did not differ between the two groups over the follow-up; a NSD resolution was demonstrated in the standard PDT group (12/13 eyes), no precise data was reported with this regard in the bevacizumab group.

## 3. Discussion

The ideal treatment of cCSC with persistent NSD lasting more than 3–6 months should effectively promote reabsorption of intraretinal fluid in a short time and reduce the number of relapses. Among the current therapeutic approaches, PDT has apparently assumed a role of preference as the first line of treatment. After an initial phase in which the PDT was used with the standard protocol and according to the settings of the TAP study, most ophthalmologists opted for a modification of the dose, fluence and time parameters to reduce the secondary effects, primarily the damage to the RPE and the occurrence of CNV. The success rates in terms of resolution of NSD are high, typically 80–100%, and seem to be independent to the protocol used, demonstrating that PDT is rather flexible in its use. In longer-term studies, the highest proportion of subjects with resolved NSD is apparently found in the subgroups receiving reduced fluence treatment. However, an increase in the number of recurrences in modified protocol studies seems to emerge and in parallel, a greater number of retreatments are also registered.

There are no consistent data on the potential toxicity of the additional effects of PDT retreatment in patients with cCSC. A punctual analysis of the effects of PDT in preserving the activity of EPR in the areas of application of the PDT spot is also complex since most of the studies do not include a precise control methodology, such as autofluorescence, as well as an uniform assessment of extrafoveal visual function by means of microperimetry is not included. We are aware microperimetry assessment is not a part of routine examination and it is quite obvious that it is a missing data in retrospective studies; however, it should be routinely performed because the BCVA is not representative of the visual function outside the foveal region and especially in the cCSC where a diffuse damage to RPE and an impairment to choroidal hyper-permeability are typical findings and the microperimetry may serve as the best indicator for subjective evaluation of retinal function. However, the reported data are comforting, since the proportion of RPE alterations secondary with atrophy or the occurrence of CNV are reported with very low incidence values.

A further limitation of the published studies is found in the retreatment criteria. Although the first treatment protocol is generally well defined, the retreatment criteria for timing and modality are quite heterogeneous, both for the NSD recurrences and for persistent NSD. Some authors discuss with patients whether to repeat the treatment or to observe, others repeat the same PDT protocol or a modified protocol, others opt for focal conventional or subthreshold laser. The paucity of studies with long follow-up further highlights this limitation; NSD recurrences are not always early and detectable in the short term and therefore this aspect of treatment is often not addressed, especially in retrospective studies.

Similar levels of effectiveness have been reported in comparative studies evaluating PDT and STLT in terms of both visual acuity improvement and NSD reabsorption.

Laser technology for subthreshold treatment is rapidly evolving; the new treatments and adapted parameters would allow the laser to be applied throughout the area of the NSD and of the RPE decompensation, also in the foveal and juxtafoveal area, contrary to what is possible with previous lasers. Therefore the conclusions of superiority of PDT over STLT reported in the PLACE trial, the largest prospective short-term study, must be cautiously interpreted.

The PLACE was widely criticized for the methodology of evaluation of the morphological and functional parameters, the design of the study, and mainly for the choice of treatment parameters for the subthreshold laser [70,71,72].

The study began recruiting in 2013 by choosing the 810 nm wavelength laser because at that time little was known about the 577 wavelength and since experimental studies had not shown significant differences between 810/532 wavelengths in histological studies. Compared with the literature available at that time, the setting had been modified in the duty cycle parameter, 5% instead of 15% as reported in previous studies, while keeping the exposure time and spot size substantially unchanged, 0.2 s and 125 microns, respectively. However, the area of application and distribution of the spots was markedly different from the reference studies. The criticisms therefore seem to converge on the hypothesis that the apparently disappointing results of the STLT in the PLACE study could be attributable to an improper laser treatment, in the methodology of the setting and in the administration of the treatment, rather than to a lack of effectiveness of the STLT itself. Finally, the lack of a detailed definition of the initial clinical findings and a precise description of the retinal architecture have been reported as missing fundamental elements in the PLACE trial necessary for an appropriate evaluation of the reported outcomes.

It is also interesting that some authors included in the PLACE trial panel have over time reported initial positive experiences in the literature in the use of STLT with 577 wavelength in patients with cCSC, treatment naive or after previous PDT treatment [73,74]. Laser technology is evolving rapidly and it is imperative to reach a broad consensus on the use of the methodology but especially on how to use this therapeutic approach in the cCSC which is a pathology with different presentation patterns, with a classification of the disease not yet available, with a variable spontaneous evolution in a variable and unpredictable time, which place a conditioning limit on the full understanding of this retinal disorder.

Limited positive experiences also exist in comparative studies of PDT with MRA drugs and with TTT but more studies with long follow-up are needed to confirm the preliminary results. On the other hand, PDT appears clearly superior to therapy with anti-VEGF drugs.

## 4. Materials and Methods

**Figure 1:**
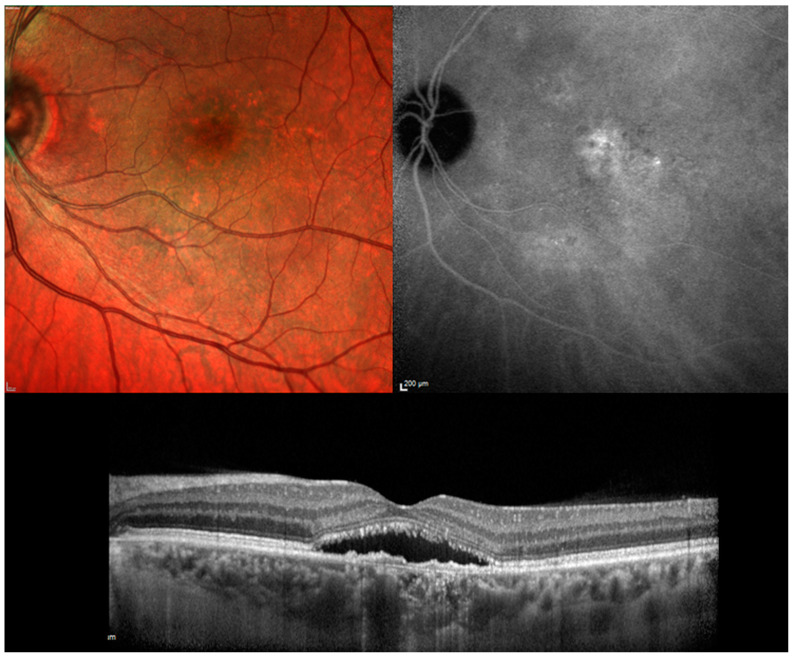
Chronic central serous chorioretinopathy. **Top Left**. Color fundus photograph: Serous neurosensory detachment at the macula associated with pigmentary mottling. **Top Right**: Indocyanine green angiography: Multiple area of hyper-fluorescence consistent with diffuse choroidal hyper-permeability are visible at the posterior pole. **Bottom Center**. Optical coherence tomography: a longitudinal scan shows the neurosensory detachment. The photoreceptor outer segments appear partially fragmented and the retinal pigment epithelium band shows an irregular profile.

**Figure 2:**
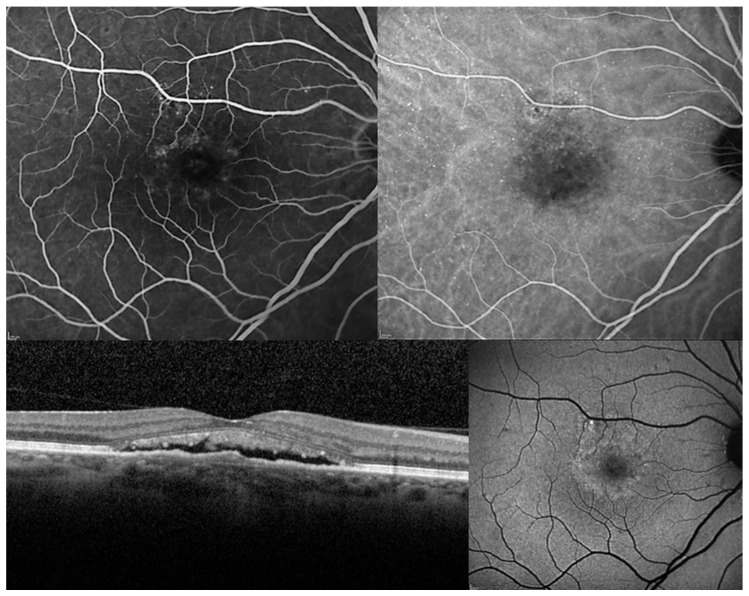
Chronic central serous chorioretinopathy. **Top Left**. Fluorescein angiography: Multiple points of retinal pigment epithelium decompensation are visible with moderate leakage at the macula. **Top Right**: Early phase of indocyanine green angiography identifies a macular neurosensory detachment and confirms a diffuse retinal pigment epitheliopathy. **Bottom Left**. Optical coherence tomography: A longitudinal scan shows a shallow neurosensory detachment with elongation of photoreceptor outer segments which appear also partially fragmented. **Bottom Right**. Fundus autofluorescence: A hyper-autofluorescence area corresponding to the macular neurosensory detachment is easily identified in association with multiple focal points of increase and reduce autofluorescence secondary to pigmentary mottling.

The authors performed an electronic search for relevant articles in PubMed from inception until August 2020. The workflow was arranged in compliance with the guidelines of the preferred reporting items for systematic reviews and meta-analyzes (PRISMA) model. Main keywords used in combination included “chronic central serous chorioretinopathy” and “photodynamic therapy with verteporfin”. Moreover, secondary keywords as “subthreshold laser treatment”, “antagonists of mineralocorticoid receptors”, “anti-VEGF drugs” and “transpupillary thermotherapy” were included in combination to find out comparative studies. Chronic CSC was defined according to the following criteria: Symptoms of subretinal fluid persisting more than three months; presence of a detachment of the neurosensory retina from the macula caused by idiopathic or diffuse leakage from the RPE and confirmed on OCT examination; leakage from the RPE detected by fluorescein angiography; choroidal vascular hyper-permeability detected on indocyanine green angiography (Figure 1 and Figure 2). All images were acquired by our department (Heidelberg HRS, Heidelberg, Germany). All images were acquired by our department (Heidelberg HRS, Heidelberg, Germany). 

Two authors (P.I. and S.D.P) independently evaluated the preliminary results of the research using the title of the manuscript and the abstract as discriminating elements.

A first exclusion was applied for review paper, case reports or small case series, articles not written in English. However, the pilot studies published in the first 3 years from the first articles published with the topic PDT/cCSC were considered. The resulting references were evaluated in order to identify relevant studies, focusing especially on randomized clinical trials, prospective studies, comparative studies and retrospective studies with large samples.

The final list produced by the two authors was then collectively evaluated and discussed with the other authors, assessing the relevance of the studies with the aims of the current research; in particular, all the authors were aware that there was no claim to produce a meta-analysis but to provide a practical review of the literature currently available, with the aim of offering points of discussion and personal critical review.

The first selection identified 402 manuscripts from which papers were excluded for: Case report (79 papers), review (41 papers), non English (31 papers), the topic was the acute form of CSC (46 papers).

Finally, for the purposes of the current research, 67 papers were included, divided with the following categorized topics: Mechanism of action and early changes after PDT administration, Standard PDT, HALF-dose PDT, HALF-fluence or Reduced fluence PDT, PDT vs. subthreshold Laser treatment, PDT vs. TTT, PDT vs. Mineralcorticoids, PDT vs. anti-VEGF.

## 5. Conclusions

To conclude, PDT definitely plays a decisive role in the first lines of treatment of cCSC. The complex pathophysiology remains to be completely determined as well as the role of alternative therapies and their possible synergistic use. It is clearly evident the need to set up the studies with a prospective design to fully understand the effects and safety of the different treatments, and especially for a better definition of the treatment and re-treatment protocols. In addition, the best option would be to evaluate the efficacy of any therapy in comparison to an untreated control group. Spontaneous remission of NSD can lead us to misjudge the results, as recently presented in the VICI trial [75].

## Figures and Tables

**Table 1:** Standard PDT for chronic central serous chorioretinopathy. Summary of studies with at least 12-month follow-up.

Authors (P/R)	F-UP	Number of Patients	BCVA	RS (dB)	NSD Resolution (%)	NSD Recurrences	Number of Treatment	Side Effects
Iacono et al. [11] [P]	1 yr	19	+14.4 letters	+2.69	95%	0	1	0
Ruiz-Del-Tiempo et al. [17] [R]	1 yr	75	+0.23 LogMar	-	93.7%	0	1–2	0
Noh et al. [18] [R]	1 yr	52	-	-	100%	0	1	Atrophy 3.85–11.59%
Silva et al. [19] [R]	4 yrs	46	+ 8 letters	-	93.4%	1	1.08	0
Moreno et al. [20] [R]	1 yr	72	+0.16 LogMar	-	100%	2	1	2 CNV, 9 RPE hyperplasia
Tarantolas et al. [10] [R]	21 mos	13	+0.07 LogMar	-	81%	3	1–2	2 CNV
Copete et al. [21] [R]	1 yr	25	+0.15 LogMar	-	100%	0	1	-
Reibaldi et al. [22] [P]	1 yr	19	+0.18 LogMar	+2.5	79%	0	1	-
Vasconcelos et al. [23] [R]	5 yrs	15	+8.4 letters	-	100%	1 (after 2 and 7 years)	1.1	-
Shin et al. [24] [R]	13 mos	33	+0.25 LogMar	-	100%	0	1	0
Ladas et al. [25] [P]	3 yrs	24	+0.31 LogMar	-	100%	2	1.3	RPE atrophy (“some”)
Sakalar et al. [26] [P]	1 yr	17	+0.22 LogMar	-	100%	0	1	0
Tsakonas et al. [27] [R]	1 yr	17	+0.4 LogMar	-	100%	1	1.2	-

Legend. P/R: Prospective/retrospective. BCVA: Best-corrected visual acuity. RS: Retinal sensitivity. NSD: Neurosensory detachment.

**Table 2:** Half-dose PDT for chronic central serous chorioretinopathy. Summary of studies with at least 12-month follow-up.

Authors (P/R)	F-UP	Number of Patients	BCVA	RS (dB)	NSD Resolution	NSD Recurrences	Number of Treatment	Side Effects
Chan et al. [30] [P]	1 yr	48	+0.16 LogMar	-	89.6%	4 (8%)	1–2	0
Dhirani et al. [31] [R]	19.3 mos	45	+0.10 LogMar	-	91%	8 (9%)	1	-
Koytarc et al. [32] [R]	1 yr	8	+0.39 LogMar	-	75%	0	1.25	1 RPE atrophy
Fujita et al. [33] [P]	1 yr	14	+0.16 LogMar	-	84%	0	1	-
Fujita et al. [34] [R]	1 yr	45	+0.15 LogMar	-	98%	2 (5%)	1–2	0
Fujita et al. [34] [R]	1 yr	204	+0.12 LogMar	+5.23 dB	89.2%	12 (5%)	1–2	1 PCV
Haga et al. [36] [R]	3 yrs	79	+0.13 LogMar	-	Estimated 98–100%	10 (12%)	1–2	-
Karakus et al. [37] [P]	25 mos	27	+0.12 LogMar	-	100%	2 (6%)	1–2	0
Kinoshita et al. [38] [R]	1 yr	29	+0.12 LogMar	-	96.5%	0	1	-
Lai et al. [39] [R]	36 mos	136	+0.21 LogMar	-	97.1%	9 (6.6%)	1–2	5 RPE atrophy,1 RPE rip
Oiwa et al. [29] [R]	1 yr	14	+10.1 letters	-	92%	1 (6%)	1	-
Tseng et al. [40] [R]	55 mos	56	+0.23 LogMar	-	100%	4 (6%)	1–2	1 RPE-atrophy, 2 CNV

Legend. P/R: Prospective/retrospective. BCVA: Best-corrected visual acuity. RS: Retinal sensitivity. NSD: Neurosensory detachment.

**Table 3:** Half-fluence PDT for chronic central serous chorioretinopathy. Summary of studies with at least 12-month follow-up.

Authors (P/R)	F-UP	Number of Patients	BCVA	RS (dB)	NSD Resolution	NSD Recurrences	Number of Treatment	Side Effects
Park et al. [42] [P]	1 yr	15 (30% F) 16 (40% F) 17 (50% F)	+0.14 +0.15 +0.21 Logmar	-	80% 94% 100%	7 (50%) 4 (25%) 0	1–2	0 0 0
Inoue et al. [41] [P]	15.5 mos	32	+0.11 decimal unit	-	88%	7 (24%)	1–2	1 CNV
Matuskova et al. [43] [R]	12 mos	32	+0.18 LogMar	-	100%	2 (6.3%)	1–2	0
Rouvas et al. [44] [R]	20 mos	29	+0.37 LogMar	-	100%	4 (13%)	1–2	0
Smretschnig et al. [45] [R]	12 mos	20	+5 letters	-	100%	3 (25%)	1–2	0

Legend. P/R: Prospective/retrospective. BCVA: Best-corrected visual acuity. RS: Retinal sensitivity. NSD: Neurosensory detachment. CNV: Choroidal neovascularization. F: Fluence.

**Table 4:** Adapted PDT protocol for chronic central serous chorioretinopathy. Summary of studies with at least 12-month follow-up and comparative design.

Authors (P/R)	Protocol	F-UP	Number of Patients	BCVA	NSD Resolution	NSD Recurrences	Number of Treatment	Side Effects
Son et al. [48] [R]	S-PDT	3 yrs	37	+0.18 L	100%	0	1	2 RPE-A
	HF-PDT		30	+0.18 L	100%	0	1	2 RPE-A
Oh et al. [52] [R]	S-PDT	1 yr	25	+0.19 L	100%	0	1	0
	HF-PDT		43	+0.12 L	100%	2	1	0
Shin et al. [24] [R]	S-PDT	13 mos	33	+0.25 L	100%	0	1	0
	HF-PDT		34	+0.17 L	94%	1	1	0
Reibaldi et al. [49] [P]	S-PDT	1 yr	18	+0.16 L	79%	2	1	2 CNV/RPE-A
	HF-PDT		23	+0.30 L	91%	1	1	0
Peng et al. [46] [R]	HD-PDT	1 yr	36	+0.11 L	94.4%	2	1	2 CNV
	HT-PDT		17	+0.17 L	91.1%	2	1	0
Liu et al. [50] [R]	HD-PDT	14.8 mos	35	+0.25 L	91%	3	1–2	0
	HT-PDT		26	+0.15 L	100%	2	1–2	0
Kim et al. [51] [R]	HD-PDT	1 yr	26	+0.19 L	100%	1	1–2	-
	HF-PDT		26	+0.20 L	96%	1	1–2	-
Nicolò et al. [53] [R]	HD-PDT	17 mos	29	+0.06 L	100%	1	1.17	0
	HF-PDT	15 mos	31	+0.19 L	83.9%	1-2-3	1.48	0

Legend. P/R: Prospective/retrospective. BCVA: Best-corrected visual acuity. RS: Retinal sensitivity. NSD: Neurosensory detachment. CNV: Choroidal neovascularization. RPE-A: Retinal pigment epithelium atrophy.

**Table 5:** Summary of comparative studies with adapted PDT protocol versus subthreshold laser treatment (STLT) for chronic central serous chorioretinopathy.

Authors (P/R)	Protocol	F-UP	N° pts	BCVA	NSD Resolution	NSD Recurrences	N° Treatment	Side Effects
Scholz et al. [54] [R]	HD-PDT	6 weeks	58	+0.04 L	21%	-	1–2	1 CNV
	STLT-577 nm		42	+0.08 L	36%	-	1–2	0
Ntomoka et al. [57] [R]	HF-PDT	6 mos	22	+0.1 L	21.7%	-	1	0
	STLT-577 nm		23	+0.2 L	59%	-	1	0
Ozmert et al. [55] [R]	HF-PDT	1 yr	18	+4 L	72%	1	1–2	0
	STLT-Microsecond		15	+4 L	80%	2	1	1 Hypofluorescent spot
Roca et al. [56] [R]	HD-PDT	17.4 mos	67	+0.03 L	95.5%	-	1 2 (5 eyes) 3 (1 eye)	1 CNV
	STLT-577 nm	15.8 mos	92	+0.21 L	92.4%	-	1 2 (12 eyes) 3 (2 eyes) 4 (2 eyes)	0
Ho et al. [59] [P]	HD-PDT	6 mos	15	+0.14 L	-	-	1	-
	STLT-577 nm		18	+0.20 L	-	-	1	-
PLACE trial [60] [P]	HD-PDT	7–8 mos	67	+6.7 L	67.2%	4 (5%)	1–2	0
	STLT-810nm		66	+4.4 L	28.8%	1 (1.3)	1–2	0

Legend. P/R: Prospective/retrospective. BCVA: Best-corrected visual acuity. RS: Retinal sensitivity. NSD: Neurosensory detachment. CNV: Choroidal neovascularization. RPE-A: Retinal pigment epithelium atrophy.
